# The relationship between smoking cessation history and significant liver fibrosis among the population with metabolic dysfunction-associated steatotic liver disease in the United States

**DOI:** 10.1371/journal.pone.0320573

**Published:** 2025-04-01

**Authors:** Zhongtao Li, Hao Guo, Hongyu He, Shu Wang, Shufen Pei, Liang Xie

**Affiliations:** 1 Department of General Surgery (Wenhua Road Campus), The Affiliated Hospital of North Sichuan Medical College, Nanchong, China; 2 Institute of Hepatobiliary Pancreatic and Intestinal Diseases, North Sichuan Medical College, Nanchong, China; 3 Department of Urology Surgery, The Affiliated Hospital of North Sichuan Medical College, Nanchong, China; 4 Department of Otolaryngology-Head and Neck Surgery, Lanzhou University Second Hospital, Lanzhou, Gansu, China; Kaohsiung Medical University, TAIWAN

## Abstract

**Background:**

Smoking was identified as a risk factor for the development of liver fibrosis in patients with metabolic dysfunction-associated steatotic liver disease (MASLD). However, the association between smoking cessation history and the development of liver fibrosis remains unclear. This study was intended to analyze the association between smoking cessation history and significant liver fibrosis in adult MASLD participants in the United States.

**Methods:**

This study utilized data from 2643 patients with MASLD from the National Health and Nutrition Examination Survey (NHANES). Significant liver fibrosis was detected based on transient elastography measurements. According to the smoking questionnaire data, patients were categorized as non-smokers, ex-smokers and current smokers. A multivariate logistic regression analysis, adjusted for weights, was performed to investigate the relationship between smoking cessation history and the presence of significant liver fibrosis in participants with MASLD.

**Results:**

A total of 2643 patients with MASLD were included in this study. Compared with non-smokers, ex-smokers had a slightly elevated risk of developing significant liver fibrosis (OR: 1.07, 95% CI: 1.02–1.13). Specifically, a positive correlation was observed between patients who quit smoking for < 20 years and significant liver fibrosis (OR: 1.07, 95% CI: 1.01–1.15). Furthermore, MASLD patients who started regularly smoking at an age of ≤ 20 years (OR: 1.09, 95% CI: 1.02–1.16) and had a smoking duration of ≥ 10 years before quitting (OR: 1.10, 95% CI: 1.02–1.18) were also highly correlated with an increased likelihood of developing significant liver fibrosis.

**Conclusions:**

This study revealed that individuals with MASLD who have ceased smoking exhibit an elevated risk for significant liver fibrosis when compared to those who never smoked. It is highly emphasized that MASLD patients who quit smoking for < 20 years, started regularly smoking at an age of ≤ 20 years, and had a smoking duration of ≥ 10 years before quitting should be extremely vigilant regarding the risk of significant liver fibrosis.

## Introduction

Metabolic dysfunction-associated steatotic liver disease (MASLD), a preferred term for non-alcoholic fatty liver disease (NAFLD) since June 2023, is a commonly occurring chronic hepatic condition[1]. It is characterized by a global prevalence estimated at 25%, affecting nearly two billion individuals worldwide[1–4]. The disease spectrum of MASLD is broad, beginning with simple fatty liver and potentially advancing to metabolic dysfunction-associated steatohepatitis (MASH), then further evolving to cirrhosis associated with MASH. In some cases, it progresses to hepatocellular carcinoma[5]. It is estimated that between 20% to 30% of MASLD patients will progress to MASH, and approximately one-fifth of MASH patients will eventually develop cirrhosis and/or hepatocellular carcinoma (HCC) [6–8]. It is anticipated that MASLD will consume a substantial amount of public health resources and place a heavy economic burden on individuals. Given this situation, it is imperative to manage it without delay[9].

Research on the factors closely related to a high likelihood of liver fibrosis in patients with MASLD mainly focused on evaluating demographic characteristics, individual behaviors, and biochemical data. The literature indicated that smoking, age, type 2 diabetes, and obesity were related to the severity of liver fibrosis in patients with MASLD[10–13]. However, data regarding the potential influence of smoking cessation history on significant liver fibrosis in individuals with MASLD was extremely limited. Moreover, it was unknown how the risk of significant liver fibrosis in MASLD patients varied with the passage of time after quitting smoking. A study in NEJM Evidence demonstrated that quitting smoking can remarkably lower the mortality rate of smokers, particularly for those who had quit smoking for more than 10 years, approaching the mortality rate of never smokers[14]. Additionally, other studies showed that quitting smoking had a significant ameliorative effect on depression, anxiety, and respiratory diseases[15,16].

Therefore, this study utilized the data of participants with MASLD from NHANES covering the period from 2017 to March 2020. The main emphasis was on evaluating the relationship between smoking cessation history and significant liver fibrosis.

## Materials and methods

### Data

This study utilized the cross-sectional data from the National Health and Nutrition Examination Survey (NHANES) database spanning from 2017 to March 2020, which had previously obtained approval from the National Center for Health Statistics (NCHS) Research Ethics Review Board (ERB), and all participants provided written informed consent. Consequently, no additional ethical review was required for our research. The NHANES gathers data on numerous health-related aspects, such as physical examinations, laboratory tests, dietary surveys, and questionnaire inquiries, which provides an intuitive reference for evaluating the health and nutrition of Americans.

### Study population

**Fig 1:**
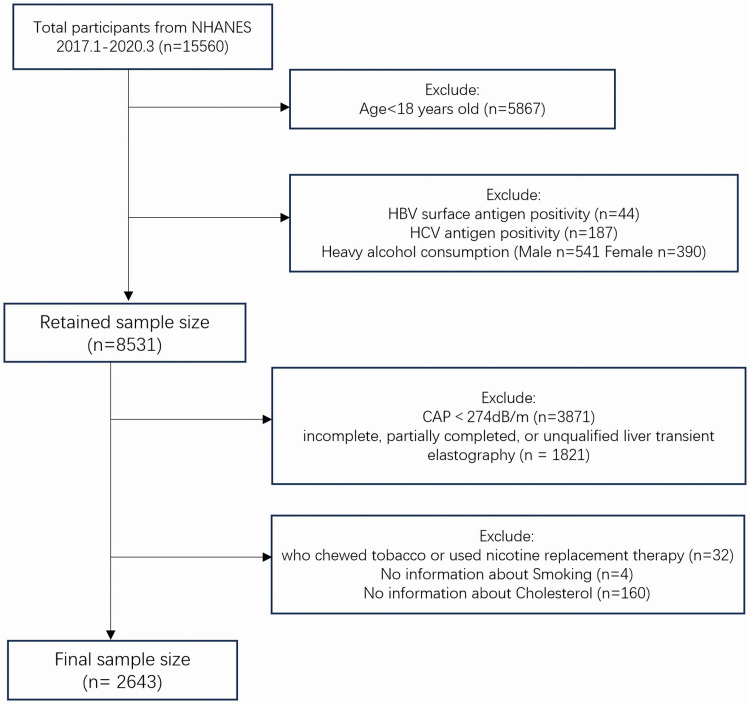
Flowchart of participants’ selection.

Among the 15560 survey participants, we excluded those under the age of 18 (n =  5,867). Additionally, we excluded participants who were positive for hepatitis B surface antigen (n =  44) and hepatitis C antibody (n =  187). Participants who consumed a large amount of alcohol were also excluded, including males with alcohol consumption exceeding 30 g/d (n =  541) and females with alcohol consumption exceeding 20 g/d (n =  390). The controlled attenuation parameter (CAP), obtained via vibration controlled transient elastography (VCTE), could define hepatic steatosis[10]. Since a median CAP of ≥  274 dB/m was regarded as a marker of steatosis[17], participants who were not diagnosed with fatty liver (CAP <  274) (n =  3871) were excluded. Additionally, based on the liver elastography status code (LUAXSTAT), we further excluded participants with incomplete, partially completed, or unqualified liver transient elastography (n =  1821)[10]. Participants who chewed tobacco and used nicotine substitutes (n =  32) were also excluded to ensure the consistency and specificity of the study population. Moreover, participants lacking data on smoking information (n =  4) and cholesterol information (n =  160) were excluded. Finally, the remaining 2643 participants were included in the study. See Fig 1 for the flowchart.

### Diagnosis of MASLD and significant liver fibrosis

Fibro Scan® is an advanced piece of equipment capable of precisely assessing hepatic steatosis and fibrosis. It utilizes the VCTE to obtain two physical parameters: the Controlled Attenuation Parameter (CAP) and the Liver Stiffness Measurement (LSM)[18]. In the study, MASLD was defined as CAP ≥ 274 dB/m, excluding individuals with viral hepatitis or excessive alcohol use[17,19,20]. The LSM, ranging from 1.5 kPa to 75 kPa, is regarded as an indicator of liver fibrosis, with larger values suggesting more severe liver fibrosis. We defined significant liver fibrosis as LSM ≥  8.0 kPa[21].

### Smoking behavior

In our study, the smoking behavior of participants was differentiated according to the cigarette usage information in the questionnaire data[22]. Specifically, based on the questionnaires which asked, “Smoked at least 100 cigarettes in life” and “Do you now smoke cigarettes?”, we categorized the participants into self-reported “current smokers”, “ex-smokers” and “non-smokers”.

The data on the duration of smoking cessation was obtained through a questionnaire titled “How long since quit smoking cigarettes”[22]. Then, we classified ex-smokers into three groups according to the duration of smoking cessation: ≤  19 years, 20 - 39 years, and ≥  40 years.

Subsequently, the age when ex-smokers first started smoking regularly was obtained through a survey question on “Age started smoking cigarettes regularly”[23]. Ex-smokers were divided into three groups according to their age of regular smoking: ≤  20 years old, 21-30 years old, and > 30 years old.

Finally, the cumulative smoking duration data of ex-smokers before quitting smoking were calculated as follows: Duration of smoking before quitting=Current age−Age started smoking cigarettes regularly−Smoking cessation duration [23]. Ex-smokers were classified into three groups according to the duration of smoking: < 5 years, 5 - 9years, and ≥  10 years.

### Covariates

Models accounted for several covariates, namely age, gender, race, physical activity, overweight, diabetes, hypertension, alcohol use and hyperlipidemia. Firstly, age, gender, and race were demographic factors. According to the American Physical Exercise Guide, adults should engage in at least 75 to 150 minutes of high-intensity exercise or 150 to 300 minutes of moderate-intensity exercise per week, or an equivalent combination of the two[24]. As a result, physical activity was classified as not meeting the guidelines (insufficient), meeting the guidelines (sufficient), or exceeding the guidelines (excessive). Overweight was determined by a body mass index (BMI) that was equal to or greater than 25 kg/m²[25]. Diabetes was defined as having a plasma fasting blood glucose level of ≥ 126mg/dL or a glycosylated hemoglobin level of ≥ 6.5%[26]. The definition of hypertension was delineated as follows: After a participant had rested tranquilly for 5 minutes, either the systolic blood pressure attained or exceeded 140 mmHg or the diastolic blood pressure reached or surpassed 90 mmHg[27]. Hyperlipidemia was diagnosed when any one of the following criteria was met: total cholesterol (TC) ≥ 200 mg/dL, triglycerides (TG) ≥ 150 mg/dL, low-density lipoprotein cholesterol (LDL-C) ≥ 130 mg/dL, or high-density lipoprotein cholesterol (HDL-C) ≤ 40 mg/dL for men and ≤ 50 mg/dL for women. Whether participants drank alcohol was determined based on their alcohol consumption data. Notably, individuals who engaged in heavy drinking were excluded from the study.

### Statistical analysis

To accurately reflect the characteristics of the national population distribution and ensure a close match with the overall population, weighting was employed in this study’s dataset[28]. Regarding the general characteristics of the research group, continuous variables were showcased as mean plus or minus standard deviation. As for categorical variables, they were presented in terms of quantity and corresponding percentage. To further probe into the relationship between smoking cessation history and significant liver fibrosis, we adjusted for covariates and constructed three weighted multiple logistic regression models. Specifically, Model 1 represented the scenario where no adjustments were made to covariates. Model 2 was adjusted in response to the significant parameters observed in the univariate analysis, while Model 3 was adjusted for all covariates. Furthermore, a more comprehensive and in-depth subgroup analysis of smoking cessation behavior was carried out, with consideration of factors such as the duration of smoking cessation, the age when ex-smokers first started smoking regularly, and the duration of smoking before quitting. All statistical analyses were consummated by utilizing R (version 4.4.1).

## Results

### Participant characteristics

Ultimately, a total of 2643 participants with MASLD were included in this study. The results of participant characteristics are presented in Table 1. Among them, 2192 individuals (83.77%) were classified as having no significant liver fibrosis associated with MASLD, while 451 individuals (16.23%) were classified as having significant liver fibrosis associated with MASLD. There were 1392 males (54.37%) and 1251 females (45.63%), with a weighted average age of 51.00 years. Additionally, 360 people (12.24%) were current smokers, 683 people (27.59%) were ex-smokers, and 1600 people (60.17%) were non-smokers. We observed that the prevalence of significant liver fibrosis was higher among participants with overweight and diabetes.

**Table 1:** General characteristics of the participants.

Variables	Overall N = 2643	Non-significant liver fibrosis N = 2192(83.77)	Significant liver fibrosis N = 451(16.23)	P-value
Age (years)	51.00 (0.71)	50.72 (0.73)	52.45 (1.13)	0.142
Sex				0.663
Male	1392 (54.37)	1143 (54.06)	249 (55.94)	
Female	1251 (45.63)	1049 (45.94)	202 (44.06)	
Race/ethnicity				0.956
Non-Hispanic White	927 (60.98)	762 (60.69)	165 (62.46)	
Non-Hispanic Black	529 (8.50)	436 (8.58)	93 (8.11)	
Mexican American	461 (12.54)	384 (12.61)	77(12.18)	
Other Race	438(10.50)	368 (10.54)	70(10.28)	
Other Hispanic	288 (7.48)	242 (7.58)	46 (6.97)	
Overweight				<0.001
No	313 (9.77)	293 (11.10)	20 (2.93)	
Yes	2309 (90.23)	1883 (88.90)	426 (97.07)	
Physical activity				0.265
Insufficient	1107 (38.62)	906 (37.91)	201 (42.33)	
sufficient	341 (14.20)	282 (13.95)	59 (15.45)	
excessive	1185 (47.18)	995 (48.14)	190 (42.22)	
Hypertension				0.354
No	1793 (78.87)	1495 (79.34)	298 (76.48)	
Yes	635 (21.13)	515 (20.66)	120 (23.52)	
Diabetes				<0.001
No	1938 (78.25)	1681 (81.98)	257 (58.98)	
Yes	705(21.75)	511 (18.02)	194 (41.02)	
Hyperlipidemia				0.950
No	783 (27.96)	655 (27.99)	128 (27.79)	
Yes	1860 (72.04)	1537 (72.01)	323 (72.21)	
Alcohol use				0.084
No	2196 (89.45)	1805 (88.80)	391 (92.69)	
Yes	243 (10.55)	212 (11.20)	31 (7.31)	
Smoking Behavior				0.001
Nonsmoker	1600 (60.17)	1343 (61.40)	257 (53.79)	
Ex-smoker	683 (27.59)	535 (25.71)	148 (37.30)	
Current smoker	360 (12.24)	314 (12.89)	46 (8.91)	

shown are numbers (%) or mean (SD). P-values are derived using either Student’s t-test or Chi-square test.

### Relationship between smoking behavior and significant liver fibrosis

Table 2 presented the results of the univariate analysis, demonstrating an association between overweight, smoking behavior, and diabetes with significant liver fibrosis.

**Table 2:** The relationship between significant liver fibrosis risk and covariates.

Variables	OR (95%CI)	P-value	Variables	OR (95%CI)	P-value
**Age (years)**	1.00 (1.00,1.00)	0.102	**Physical activity**		
**Sex**			Insufficient	1.00 (reference)	
Male	1.00 (reference)		sufficient	1.00 (0.93,1.07)	0.972
Female	0.99 (0.94,1.04)	0.661	excessive	0.97 (0.92,1.02)	0.174
**Race/ethnicity**			**Hypertension**		
Non-Hispanic White	1.00 (reference)		No	1.00 (reference)	
Non-Hispanic Black	0.99 (0.93,1.06)	0.844	Yes	1.02 (0.97,1.08)	0.377
Mexican American	1.01 (0.96,1.06)	0.719	**Diabetes**		
Other Race	1.00 (0.95,1.05)	0.917	No	1.00 (reference)	
Other Hispanic	1.00 (0.94,1.06)	0.965	Yes	1.20 (1.13,1.28)	<0.001
**Overweight**			**Alcohol use**		
No	1.00 (reference)		No	1.00 (reference)	
Yes	1.13 (1.08,1.19)	<0.001	Yes	0.94 (0.89,1.00)	0.051
**Smoking Behavior**			**Hyperlipidemia**		
Nonsmoker	1.00 (reference)		No	1.00 (reference)	
Ex-smoker	1.08 (1.02,1.13)	0.007	Yes	1.00 (0.96,1.05)	0.950
Current smoker	0.97 (0.93,1.02)	0.219			

Models were unadjusted.

The relationship between smoking behavior and significant liver fibrosis was presented in Table 3 through weighted multiple logistic regression analysis results. Our research findings indicated that in Model 1 without covariate adjustment (OR, 1.08; 95% CI: 1.02 to 1.13), Model 2 adjusted for overweight, diabetes (OR, 1.06; 95% CI: 1.01 to 1.12), and Model 3 adjusted for all covariates (OR, 1.07; 95% CI: 1.02 to 1.13), a positive correlation was detected between ex-smokers and the likelihood of significant liver fibrosis in those diagnosed with MASLD. Surprisingly, however, no association was found between current smokers and significant liver fibrosis.

**Table 3:** Relationship between smoking behavior and significant liver fibrosis.

Variable	Significant liver fibrosis(LSM ≥ 8.0 kPa)					
	Model 1^a^		Model 2^b^		Model 3^c^	
	OR (95%CI)	P-value	OR (95%CI)	P-value	OR (95%CI)	P-value
**Smoking Behavior**						
Nonsmoker	1.00 (reference)		1.00 (reference)		1.00 (reference)	
Ex-smoker	1.08 (1.02,1.13)	0.007	1.06 (1.01,1.12)	0.013	1.07 (1.02,1.13)	0.012
Current smoker	0.97 (0.93,1.02)	0.219	0.98 (0.94,1.02)	0.310	0.97 (0.93,1.02)	0.211

^a^Model 1 was unadjusted.

^b^Model 2 was adjusted for overweight, diabetes.

^c^Model 3 was adjusted for age, gender, race/ethnicity, overweight, physical activity, hypertension, diabetes, alcohol use, hyperlipidemia.

Abbreviation: CI = confidence interval; OR = odds ratio.

### Subgroup analyses

**Fig 2:**
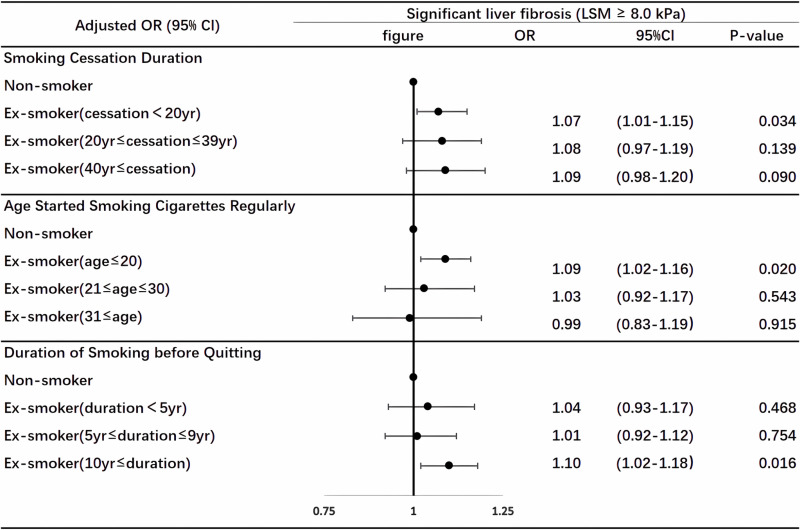
Forest plot of subgroup analysis stratified by smoking cessation duration, age started smoking cigarettes regularly, and duration of smoking before quitting.

Table 4 and Fig 2 displayed the results of stratified subgroup analysis on the relationship among the duration of smoking cessation, the age when ex-smokers first started smoking regularly, and the duration of smoking before quitting in ex-smokers. We discovered that ex-smokers with a shorter smoking cessation duration, a younger age of onset of frequent smoking, and a longer duration of smoking before quitting were more likely to have an association with significant liver fibrosis. In the fully adjusted Model 3, ex-smokers who quit smoking for ≤  19 years (OR: 1.07, 95% CI: 1.01–1.15) showed a statistically significant correlation with significant liver fibrosis compared with non-smokers. However, as the smoking cessation time continued to increase, we found that the incidence of liver fibrosis in ex-smokers who quit smoking for 20–39 years (OR: 1.08, 95% CI: 0.97–1.19) and ex-smokers who quit smoking for ≥  40 years (OR: 1.09, 95% CI: 0.98–1.20) became statistically non-significant compared to non-smokers.

**Table 4:** Results of the subgroup analysis stratified by smoking cessation duration, age started smoking cigarettes regularly, and duration of smoking before quitting.

Variable	Significant liver fibrosis(LSM ≥ 8.0 kPa)					
	Model 1^a^		Model 2^b^		Model 3^c^	
	OR (95%CI)	P-value	OR (95%CI)	P-value	OR (95%CI)	P-value
**Smoking Cessation Duration**						
Nonsmoker	1.00 (reference)		1.00 (reference)		1.00 (reference)	
Ex-smoker (cessation < 20yr)	1.08 (1.01,1.15)	0.024	1.07 (1.01,1.14)	0.019	1.07 (1.01,1.15)	0.034
Ex-smoker (20yr≤cessation ≤ 39yr)	1.06 (0.98,1.15)	0.118	1.05 (0.97,1.13)	0.244	1.08 (0.97,1.19)	0.139
Ex-smoker (40yr≤cessation)	1.11 (0.99,1.25)	0.073	1.06 (0.96,1.17)	0.218	1.09 (0.98,1.20)	0.090
**Age Started Smoking Cigarettes Regularly**						
Nonsmoker	1.00 (reference)		1.00 (reference)		1.00 (reference)	
Ex-smoker (age ≤ 20)	1.09 (1.03,1.16)	0.008	1.07 (1.01,1.14)	0.016	1.09 (1.02,1.16)	0.020
Ex-smoker (21 ≤ age ≤ 30)	1.01 (0.92,1.12)	0.760	1.00 (0.91,1.11)	0.960	1.03 (0.92,1.17)	0.543
Ex-smoker (31 ≤ age)	0.95 (0.81,1.12)	0.548	0.97 (0.82,1.14)	0.679	0.99 (0.83,1.19)	0.915
**Duration of Smoking before Quitting**						
Nonsmoker	1.00 (reference)		1.00 (reference)		1.00 (reference)	
Ex-smoker (duration < 5yr)	1.01 (0.92,1.11)	0.814	1.02 (0.92,1.13)	0.681	1.04 (0.93,1.17)	0.468
Ex-smoker (5yr≤duration ≤ 9yr)	1.02 (0.94,1.11)	0.597	1.01 (0.93,1.11)	0.771	1.01 (0.92,1.12)	0.754
Ex-smoker (10yr≤duration)	1.10 (1.02,1.18)	0.011	1.08 (1.01,1.15)	0.027	1.10 (1.02,1.18)	0.016

^a^Model 1 was unadjusted.

^b^Model 2 was adjusted for overweight, diabetes.

^c^Model 3 was adjusted for age, gender, race/ethnicity, overweight, physical activity, hypertension, diabetes, alcohol use, hyperlipidemia.

Abbreviation: CI = confidence interval; OR = odds ratio; yr = years.

In addition, ex-smokers who started regularly smoking at an age ≤  20 years (OR: 1.09, 95% CI: 1.02-1.16) were more likely to have a strong association with significant liver fibrosis compared to non-smokers. No significant correlation was observed among ex-smokers who started regularly smoking at an age of 21-30 (OR: 1.03, 95% CI: 0.92-1.17) and ex-smokers who started regularly smoking at an age of ≥  31 years (OR: 0.99, 95% CI: 0.83-1.19).

Finally, upon analyzing the duration of smoking before quitting, it was discovered that ex-smokers who had smoked for ≥  10 years before quitting were more likely to have a strong association with significant liver fibrosis compared to non-smokers (OR: 1.10, 95% CI: 1.02 - 1.18). However, no such association was observed among ex-smokers with a smoking duration of 5-9 years (OR: 1.01, 95% CI: 0.92 - 1.12) or those with less than 5 years of smoking duration (OR: 1.04, 95% CI: 0.93 - 1.17).

## Discussion

Metabolic dysfunction-associated steatotic liver disease (MASLD) has emerged as a global health challenge, afflicting numerous populations[29]. Patients with advanced fibrosis are particularly prone to serious health hazards such as liver dysfunction and hepatocellular carcinoma. Tobacco contains at least 79 carcinogens, which can exert various short-term and long-term adverse effects on the human body[30]. Relevant studies have indicated that smoking increases the risk of liver fibrosis in patients who have MASLD[10]. However, there are currently very few articles reporting the association between smoking cessation and significant liver fibrosis. In this nationally representative sample of adult MASLD patients in the United States, this study uncovered a statistically significant positive association between smoking cessation history and the risk of significant liver fibrosis. Even after we accounted for known confounding factors including gender, age, race, physical activity level, overweight, diabetes, alcohol use, hyperlipidemia, and hypertension, this positive correlation remained.

The benefits of quitting smoking for smokers are almost immediate, regardless of when they choose to quit. These benefits encompass lowering blood pressure and reducing the risks related to cardiovascular diseases and cancer[31]. When grouped according to the smoking cessation duration, we discovered that the incidence rate of significant liver fibrosis in ex-smokers who quit smoking for ≤ 19 years was statistically significantly positive correlated with that of non-smokers. This might be attributed to the long-term harm caused by nicotine and tar to the body[32]. Even after quitting smoking, it takes a certain amount of time to repair the damage within the body[33]. As reported by Ding et al., smoking heightens the risk of peripheral arterial disease (PAD), and this elevated risk can persist for up to 30 years even after quitting[34]. However, the incidence rate of significant liver fibrosis in ex-smokers who had quit smoking for ≥ 20 years was not statistically related to that in non-smokers. This implies that the benefits of quitting smoking can accumulate over time[35]. The liver possesses a certain degree of self-healing ability[36], and after a long period of smoking cessation, the liver may have basically recovered to a state similar to that of non-smokers. Consequently, the probability of significant liver fibrosis in ex-smokers at this point is not substantially different from that of non-smokers. Additionally, quitting smoking can increase the overall lifespan of smokers and reduce the risk of premature death. Studies have demonstrated that individuals who quit smoking between the ages of 25 and 34 can expect an overall lifespan increase of approximately 10 years[31].

At the same time, we have also discovered a robust positive correlation between MASLD participants who had started frequent smoking at the age of ≤  20 and significant liver fibrosis. This might be because for adolescents aged 20 or younger, their bodies are still in the process of development and their livers are more sensitive to harmful substances in tobacco[37]. Moreover, handling harmful substances like nicotine and tar imposes a greater metabolic burden on the not yet fully matured liver, ultimately resulting in an increased occurrence of significant liver fibrosis among this population. Additionally, MASLD participants who had a smoking duration of ≥  10 years before quitting had a significantly elevated probability of developing significant liver fibrosis. This finding further bolsters the view that the damage caused by smoking to the liver is a gradual accumulation process[32]. Moreover, this kind of damage will still be manifested to a certain extent even after quitting smoking[33]. This implies that the harm caused to the liver by past smoking behavior does not vanish immediately upon quitting. Instead, it may linger in the body for a period of time and may even have long-term impacts on liver health.

There exists a tight association between smoking and hepatic steatosis as well as fibrosis. Evidence indicates that smoking heightens the likelihood of advanced liver fibrosis for those with MASLD[38]. However, our study did not identify a statistically significant correlation between current smokers and significant liver fibrosis. This finding is somewhat surprising, given the well-documented detrimental effects of smoking on liver health. This result is similar to the findings of Zein et al. In their study, an association between significant smoking history and advanced liver fibrosis (stages 3-4) is demonstrated in patients with non-alcoholic fatty liver disease (NAFLD), but the direct link between current smoker and advanced liver fibrosis remains unconfirmed[39]. This could potentially stem from recall errors during the data collection phase or limitations in the precision of the analytical methods employed. Additionally, it is possible that there are undiscovered protective mechanisms at play. For example, Chen et al.‘s research demonstrated that specific gut bacteria can effectively degrade nicotine, thereby alleviating the progression of MASLD caused by smoking[40]. Such mechanisms may potentially offset some of the adverse effects of smoking on liver fibrosis. In conclusion, while our findings were unable to demonstrate a statistically significant correlation between current smokers and significant liver fibrosis, they do not negate the potential harmful effects of smoking on liver health.

Currently, there are numerous diverse theories regarding how smoking causes liver damage. Firstly, smoking activates NADPH oxidase and augments the generation of reactive oxygen species (ROS), thereby intensifying the oxidative stress process, which is a recognized damage mechanism in MASLD[41]. Additionally, smoking induces dysbiosis of the gut microbiota and bacterial translocation, and activates Toll-like receptor 4 on hematopoietic stem cells, leading to cell activation and fibrosis[42]. Smoking is also related to hypoxia. Chronic hypoxia may also contribute to the occurrence of liver fibrosis in MASLD patients[43]. A study has demonstrated that chronic intermittent hypoxia in high-fat diet mice results in liver inflammation, extensive collagen deposition, and increased lipid peroxidation as well as pro-inflammatory cytokine expression in liver tissue[43]. Moreover, smoking is closely linked to the development of many other diseases. A cross-sectional study reported that smoking fatty liver subjects had a higher frequency of developing metabolic syndrome compared to non-smoking fatty liver subjects[44]. Furthermore, smoking is also a contributing factor to the occurrence of vascular disorders and chronic obstructive pulmonary disease (COPD)[45, 46].

It was undeniable that our study had some limitations. Firstly, this study was based on cross-sectional analysis and cannot determine the causal relationship between significant liver fibrosis and smoking cessation behavior. Specifically, it was impossible to determine whether the disease had an impact on lifestyle, so further studies with higher levels of evidence were required to illuminate the association between smoking cessation behavior and significant liver fibrosis. Secondly, smoking data collected through questionnaire surveys might lead to recall bias, resulting in unreliable and inaccurate data on smoking status. Thirdly, the gold standard for diagnosing liver fibrosis was liver biopsy, and in this study, VCTE was used to measure liver stiffness. Although VCTE was superior to liver enzymes and ultrasound, it was still not as accurate as liver biopsy. Finally, although we had adjusted known confounding factors in the relationship between smoking cessation behavior and significant liver fibrosis, we could not rule out the possibility of other unidentified confounding factors.

## Conclusion

According to our research findings, compared with non-smoking participants with MASLD, ex-smoking participants with MASLD possess a relatively higher risk of presenting with significant liver fibrosis, particularly among ex-smokers who have quit smoking for ≤  19 years. Additionally, we also observed that ex-smokers who started frequent smoking at the age of ≤  20, as well as ex-smokers who had a smoking duration of ≥  10 years before quitting, also exhibited this high risk. These findings indicate that in the management of liver fibrosis, it is essential to pay attention to the above-mentioned groups, closely monitor them, and intervene at the right time to reduce the risk of patients developing severe liver disease.

## Supporting information

S1 File Summary table of detailed information for all participants.
(XLSX)
